# UKCGG Consensus Group guidelines for the management of patients with constitutional *TP53* pathogenic variants

**DOI:** 10.1136/jmedgenet-2020-106876

**Published:** 2020-06-22

**Authors:** Helen Hanson, Angela F Brady, Gillian Crawford, Rosalind A Eeles, Sarah Gibson, Mette Jorgensen, Louise Izatt, Aslam Sohaib, Marc Tischkowitz, D Gareth Evans, Consensus Group Members, Muna Ahmed

**Affiliations:** 1 St George's Hospital NHS Foundation Trust, South West Thames Regional Genetic Services, London, UK; 2 North West Thames Regional Genetics Service, Kennedy-Galton Centre, London North West University Healthcare NHS Trust, Harrow, UK; 3 Clinical Genetics, University Hospital Southampton NHS Foundation Trust, Southampton, UK; 4 Oncogenetics Team, The Institute of Cancer Research, Sutton, Surrey, UK; 5 Clinical Oncology and Oncogenetics, Royal Marsden NHS Foundation Trust, London, London, UK; 6 Peninsula Clinical Genetics, Royal Devon & Exeter Hospital, Exeter, UK; 7 Paediatric Oncology, Great Ormond Street Hospital for Children NHS Foundation Trust, London, UK; 8 Clinical Genetics, Guy's and St Thomas' NHS Foundation Trust, London, UK; 9 Radiology, Royal Marsden Hospital NHS FoundationTrust, London, UK; 10 Academic Department of Medical Genetics, University of Cambridge, Cambridge, UK; 11 Genetic Medicine, Central Manchester University Hospitals NHS FoundationTrust, Manchester, UK

**Keywords:** clinical genetics, paediatric oncology, genetics, guidelines, screening

## Abstract

Constitutional pathogenic variants in *TP53* are associated with Li-Fraumeni syndrome or the more recently described heritable *TP53-*related cancer syndrome and are associated with increased lifetime risks of a wide spectrum of cancers. Due to the broad tumour spectrum, surveillance for this patient group has been limited. To date, the only recommendation in the UK has been for annual breast MRI in women; however, more recently, a more intensive surveillance protocol including whole-body MRI (WB-MRI) has been recommended by International Expert Groups. To address the gap in surveillance for this patient group in the UK, the UK Cancer Genetics Group facilitated a 1-day consensus meeting to discuss a protocol for the UK. Using a preworkshop survey followed by structured discussion on the day, we achieved consensus for a UK surveillance protocol for *TP53* carriers to be adopted by UK Clinical Genetics services. The key recommendations are for annual WB-MRI and dedicated brain MRI from birth, annual breast MRI from 20 years in women and three-four monthly abdominal ultrasound in children along with review in a dedicated clinic.

## Background

Li-Fraumeni syndrome (LFS) is a rare inherited cancer predisposition syndrome, first described by Frederick Li and Joseph Fraumeni in 1969.^1^ LFS is caused by pathogenic variants (PVs) in the *TP53* gene.^2^ Individuals with LFS have a very substantially increased lifetime risk of cancer, with risks reported to be as high as 22% by 5 years of age, 41% by 18 years of age and approaching 100% by 70 years of age.^3 4^ The most frequent cancers that occur in patients with LFS are bone and soft tissue sarcomas, very early-onset breast cancer, malignant tumours of the central nervous system and adrenocortical cancers (ACCs). However, of note, with expanded testing of cancer predisposition genes outside the context of a strong family history of cancer and identification of *TP53* PVs in population databases, there is widening recognition that the phenotypical spectrum and cancer risk for individuals with a constitutional *TP53* PV may be both broader and lower than initially described.^5–7^ This has led to recent suggestions to expand the clinical description of constitutional *TP53* PVs from LFS to a wider cancer predisposition syndrome designated heritable *TP53-*related cancer syndrome (h*TP53*rc) (GD Evans from GENTURIS expert group, personal communication).

Most *TP53* carriers in the UK have been identified through referral to clinical genetics services due to a strong personal or family history of cancer, where genetic testing has largely been recommended on clinical criteria such as the ‘Chompret criteria’.^3^ Once a PV has been identified within a family, predictive genetic testing can be offered to at-risk relatives.

There has been a long-standing hesitancy to offer genetic testing of *TP53* due to the absence of surveillance recommendations should a *TP53* PV be identified. To date, the only nationally agreed surveillance recommendation for individuals with a *TP53* PV has been for breast surveillance with annual breast MRI from age 20 in women.^8^ However, over the past 5 years, recommendations for more comprehensive surveillance for *TP53* carriers have been made, most notably the Toronto protocol, an intensive protocol involving clinical, biochemical and radiological surveillance that has demonstrated a survival benefit.^9 10^ One of the unique aspects of this protocol is the use of whole-body MRI (WB-MRI). WB-MRI has been separately studied in a number of centres across North America, Canada and Europe, and a meta-analysis of the data from 13 studies in 578 *TP53* carriers from six countries, including the UK SIGNIFY study, evidences a 7% (95% CI 5% to 9%) new cancer detection rate, with the majority of cancers being detected at a sufficiently early stage to allow treatment with curative intent.^11 12^

In 2017, an expert international group published recommendations on surveillance for individuals with LFS.^13^ This publication necessitated a review of current UK practice. Therefore, the UK Cancer Genetics Group (UKCGG), with support from the George Pantziarka TP53 Trust (the UK LFS patient advocacy group), organised a national consensus meeting on 6 July 2018 to agree on a consistent approach to the management of *TP53* carriers across the UK.

## Methods

### Pre-meeting preparation

Preparation for the meeting included a systematic review of the literature to identify key publications on surveillance in LFS to inform discussion. As there is limited literature available on this topic, only five key publications were selected for discussion at the meeting: publications relating to the Toronto protocol, recent International Recommendations, results of a meta-analysis of baseline WB-MRI and results from a UK study of WB-MRI.^9 11–13^

Prior to the meeting, these publications and a survey (online supplementary information 1) were sent out to the 24 UK Regional Genetics centres. This approach has been successful for other UKCGG guidelines.^14^ The survey focused on questions relating to current practice and sought opinion on the surveillance recommendations made by the International *TP53* Consensus Group.^13^ The response rate to the survey was 22/24 (91.7%). The themes arising from the survey were then used to create a series of key questions to be addressed at the consensus meeting.

10.1136/jmedgenet-2020-106876.supp1 Supplementary data

### Consensus Group participants

Forty-three stakeholders attended from across the UK and Ireland, including patient support group representatives (n=3), clinical cancer geneticists (n=19), genetic counsellors (n=6), paediatric oncologists (n=2), oncologists (n=3), radiologists (n=8), psychologists (n=1) and pathologists (n=1). Each of the 24 UK genetics centres was represented.

### Presentation of current knowledge

The first part of the meeting was structured into a series of talks. The talks covered the history of LFS and *TP53* the associated cancer risks, surveillance suggested by other expert groups, a UK perspective on WB-MRI and the survey results. These lectures provided a review of the available evidence and equipped the Consensus Group, who were from a variety of backgrounds with up-to-date evidence on which to base their recommendations. The agenda and presentations from the day are available online https://www.ukcgg.org/information-education/ukcgg-consensus-meetings/

### Discussion groups

The group was then divided into three small working groups to discuss three key questions listed as follows. Participants in each group were selected to include a range of the multidisciplinary team. Each group had a nominated facilitator and scribe. After each discussion, there was feedback and discussion and debate by the wider group until a consensus was reached. Once all delegates had discussed the three key questions, the final part of the day was a group forum to agree on a UK surveillance protocol.

### Meeting report

Following the meeting, the agreed statements were circulated to the group and presented at the UK-CGG Winter Meeting 2018 for further comments to ensure that the statements were an accurate representation of the day and consensus had been reached. A summary of the meeting was posted on the UKCGG website in March 2019 (https://www.ukcgg.org/information-education/ukcgg-consensus-meetings/).

## Results

### Pre-meeting survey

Most centres who responded to the survey (17/22) were not currently offering any surveillance other than the recommended annual breast MRI from age 20 years, which was available by direct referral to the NHS Breast Screening Programme. Four centres were offering adrenal ultrasound (US) in children, and three centres were able to offer WB-MRI or were in the process of trying to set this up.

With regard to opinion on the International Consensus guidelines, the main themes that arose from survey respondents were either disagreement or concern with specific areas of the guidelines and/or the implementation of any agreed recommendations within the NHS. Specifically, there was concern about the requirement for general anaesthetic for WB-MRI in childhood and uncertainty about the requirement for brain MRI, in addition to WB-MRI and the use of gadolinium in imaging. There was also strong disagreement with the recommendation for upper gastrointestinal (GI) endoscopy and lower GI colonoscopy. More general comments were the lack of clinical evidence for benefit of early detection on long-term outcome of patients and concern over the high false-positive rate of WB-MRI.

Regarding implementation of any guidelines, survey respondents wanted to address who should be responsible for coordinating surveillance and whether this should be undertaken in all centres or in specialist clinics. The change in practice towards offering predictive testing in childhood if surveillance was available was also discussed.

The results and questions raised by the survey were then split into three main themes to be discussed on the day.

### Question 1: what surveillance should we recommend?

The group reviewed the International Consensus recommendations based on the Toronto Protocol.^13^ For the majority of the recommendations, consensus was reached that we should adopt the same recommendations. However, there was further discussion of a number of key areas; clinical breast examination, US of the abdomen and pelvis in adults, upper and lower GI endoscopy and annual dermatological examination.

Clinical breast examination has not been proven to be an effective screening tool,^15^ and the Department of Health issued advice that clinical breast examination was not an appropriate screening technique in February 1998 (PL/CMO/98/1). Instead the recommendation of the Consensus Group would be to ensure that women are educated in breast self-examination and to recommend annual breast MRI surveillance, and the option of risk-reducing mastectomy was discussed.

With regard to abdominal US in adults, it was not felt that US would offer additional benefit over WB-MRI in adults, particularly as the incidence of ACC in adults is low. In the Toronto study, while US in childhood was found to be beneficial, abdominal and pelvic US did not detect any cancers in adults.^9^

Consensus was reached not to offer surveillance of the upper GI tract. The main reason for this decision is that current data suggest that only a small number of *TP53* carriers develop an upper GI malignancy. Recent data suggest that 2% of *TP53* carriers develop stomach cancer and 0.6% develop oesophageal cancer.^3^ This risk is comparable to the general population risk of developing gastric cancer from birth to age 74 of 1.87% in men and 0.79% in women worldwide.^16^ Upper GI endoscopy is also not recommended in the Toronto protocol, and there is limited evidence to suggest that it results in any survival benefit.^17^ Although there is no specific data relating to *Helicobacter pylori* (HP) eradication in *TP53* carriers, it is recognised that HP is a risk factor for gastric cancer and evidence that eradication reduces gastric cancer risk.^18^ Therefore, the group felt that advice on testing and eradication of HP should be specifically recommended, as well as more general advice not to smoke.

Consensus was reached not to offer surveillance of the lower GI tract. Colorectal cancer has been postulated to be associated with LFS; however, review of the relevant literature provides little supporting evidence for this. Sixteen cases of colorectal cancer were reported in a series of 397 patients with cancer from 64 families with LFS, giving a similar risk to general population risk (4%), although admittedly at younger ages.^19^ Of note, many of the patients with colorectal cancer were not proven *TP53* carriers. Another study reported 6/497 *TP53* carriers in a series of patients with colorectal cancer at age 40 years or younger, but evaluation of the identified variants showed that only 1/6 variants would be classified as likely pathogenic or pathogenic based on current guidelines.^20^ Therefore, the opinion of the Consensus Group is that there is no sufficient evidence that colorectal cancer risk is increased in LFS and that colonoscopic surveillance should only be considered if there is a family history of colorectal tumours according to recently published guidelines and investigation for other inherited causes of colorectal cancer if appropriate.^21^

Regarding annual dermatological review, the group recognised the resource implications for this, and it was felt that this could be undertaken by the patient’s general practitioner with a low threshold for referral to dermatology.

The Consensus Group also felt that it was important to ensure there was detailed discussion of ‘red flag’ symptoms in both children and adults and provide information on relevant resources and to discuss the importance of making positive lifestyle choices, for example, not smoking, eating a healthy diet and being physically active.

The concern raised through the premeeting survey regarding the requirement for general anaesthetic for WB-MRI in childhood was also discussed. Data on cancer risk were reviewed, and it was strongly felt that due to the high risks of cancer reported in infancy,^3^ general anaesthetic was acceptable in order to provide the appropriate surveillance. This position was supported by the patient advocates present at the meeting.

The group also discussed the requirement for dedicated brain MRI. Malignant tumours of the central nervous system are a core cancer in LFS. The meta-analysis of WB-MRI compared the outcomes of WB-MRI with dedicated brain MRI and demonstrated that of 10 brain tumours identified in individuals having both WB-MRI and brain MRI, 5 tumours were missed by WB-MRI.^12^ The Consensus Group therefore agreed that both dedicated brain MRI and WB-MRI should be included in the surveillance protocol. The use of gadolinium was felt to be necessary for the first but not subsequent scans.

The surveillance protocol agreed by the Consensus Group is detailed in table 1.

**Table 1:** Agreed surveillance recommendations for *TP53* carriers

Tumour	Screening recommendation
ACC	Abdominal USS 3–4 monthly birth:18 years Biochemistry (17 OH-progesterone, total testosterone, DHEAS, androstenedione) should only be performed where there is an unsatisfactory USS.
Breast cancer (women only)	Annual dedicated MRI from age 20–70 years Consider risk-reducing mastectomy from age 20 years
Brain tumour	Annual dedicated brain MRI from birth (first MRI with contrast)*
Sarcoma	Annual WB-MRI† from birth*
Haematological	Routine FBC are not indicated due to lack of evidence that these detect haematological malignancy at an early stage.
Colon	Colonoscopy only indicated when family history of colorectal cancer or polyposis‡; consider investigation for, possibly coinherited, causes if strong family history of colorectal cancer or polyposis The presence of microcytic anaemia should prompt investigation for a gastrointestinal tract malignancy (routine FBC not advised).
Gastric	Recommend *Helicobacter pylori* testing and eradication if required Endoscopy not indicated due to lack of evidence
Skin	Annual dermatology review from 18 years (general practitioner or dermatology) General advice on use of high protection factor sunscreen and covering up in sun
Physical examination	Full physical examination 3–4 monthly in children (including blood pressure, anthropometric measurements, signs of virilisation and neurological exam) Routine physical examination not recommended in adults; advise detailed discussion of ‘red flag’ symptoms and low threshold for fast track referral of persistent or unusual symptoms
Other	Recommend detailed discussion of red flag symptoms in both children and adults and provide information on relevant resources. Discuss importance of making positive lifestyle choices (eg, not smoking, eating a healthy diet, limiting alcohol consumption, sun protection, keeping physically active and providing appropriate resources).

Notes: (1) Currently on most scanners, arms are not covered adequately, and these should be evaluated clinically; (2) patients to be recalled for detailed imaging to evaluate uncertain lesions; (3) units wanting to do WB-MRI have to opt in (ie, self-certify quality for WB-MRI); (4) a minimum number of scans per year in a unit have not been specified; (5) optional sequences can be performed at the discretion of the unit; (5) radiology should be informed of any current clinical symptoms to inform interpretation of scan.

*Children weighing less than 20 kg need sedation, examination without anaesthetic may be possible from the age of 5 years with help from a dedicated play specialist. Feed and wrap approach may also be possible in the first year.

†Recommended core minimum sequence for WB-MRI (adults): T1, T2 fat sat/STIR or diffusion and non-fat sat T2; images can be acquired in axial or coronal planes or mixture; slice thickness (including gap) not greater than 10 mm; coverage vertex to feet; optional sequences at the discretion of the unit. Radiology should be informed of any current clinical symptoms to inform interpretation of scan.

‡For individuals with a family history of colorectal cancer, we would suggest reference to the recently published British Society of Gastroenterology/Association of Coloproctologists of Great Britain and Ireland/United Kingdom Cancer Genetics Group guidelines for hereditary colorectal cancer.^21^

FBC, full blood count.

### Question 2: who should we offer surveillance to?

*TP53* PVs are identified in approximately 30% patients fulfilling Chompret criteria and up to 75% of patients with classic LFS.^3^ This discussion focused on whether we would offer surveillance only to patients with confirmed PVs in *TP53*, those who meet clinical criteria for LFS but do not have a confirmed *TP53* PV or those with PVs predicted to be of lower penetrance for example, the Brazilian founder PV c.1010G>A, p.ArgR337His. In addition, we also considered the situation of patients who are at 50% risk of a familial *TP53* PV but do not wish to proceed with predictive testing.

The Consensus Group felt that for patients with classic LFS but without a confirmed *TP53* PV, surveillance should be offered for patients affected with a relevant cancer. It was felt that Li-Fraumeni-like (LFL) criteria had largely been designed to broaden the clinical criteria and increase sensitivity for testing of *TP53*. Therefore, for LFL families without a *TP53* PV, the lifetime risks of cancer were unlikely to be in the range of that observed for classic LFS and surveillance of this patient group was not supported. With regards to lower-penetrance variants, it was felt that at present without the benefit of clear genotype phenotype correlations, the surveillance protocol should be offered to all patients with a *TP53* PV, recognising that more reliable phenotype–genotype data may lead to genotype-specific modifications of these recommendations in the future.

Offering the full surveillance protocol for those at 50% risk was not considered appropriate due to the intensity of the surveillance protocol. However, women at 50% of a *TP53* PV can still be offered breast MRI in line with National Institute for Clinical Excellence (NICE) guidelines.^8^ Children at 50% risk should be reviewed in a specialist clinic, ideally with a paediatric oncologist and clinical geneticist, to discuss the surveillance and testing, red flag symptoms. If predictive testing is not pursued open access back to the clinic should be offered for when the family wish to pursue testing or if they have further questions.

The Consensus Group noted that the recommendation of a surveillance protocol will likely change both clinician and patients’ perspectives of having a genetic test for *TP53* and also that it would be appropriate to offer predictive testing in childhood for a familial PV.

The recommendations for who should be offered surveillance are set out in box 1.

Box 1 **Eligibility for surveillance**

The aforementioned screening programme should be offered to

- Patients with a *TP53* PV (class 4 or 5 according to American College of Medical Genetics ACMG guidelines).
- Patients with constitutional (germline) mosaicism for a *TP53* PV (verified by confirmation in two tissues).
- Patients with low-penetrance *TP53* PVs, until further data on cancer risk available.
- Patients affected with cancer fulfilling classic Li-Fraumeni syndrome (LFS) criteria#* without a pathogenic *TP53* variant (confirmation of cancer diagnoses required).

The above screening programme should *not* be offered to

- Patients at 50% risk of familial *TP53* PV. Patients at 50% risk should have appropriate counselling and support but should be encouraged to consider testing in order to access cancer screening.
- Paediatric patients at 50% risk of familial *TP53* PV should continue to be offered support and review in a specialist clinic, but screening is not appropriate unless they are confirmed to have inherited the familial PV.
- Adult patients at 50% risk.*†

*Classic LFS criteria=proband with a sarcoma diagnosed before age 45 years and a first-degree relative with any cancer before age 45 years and a first-degree or second-degree relative with any cancer before age 45 years or a sarcoma at any age.†Women at 50% risk of a *TP53* PV can be offered annual breast MRI from age 20 years.

Patients with an LFL family history. Screening should be offered on the basis of cancer in the family according to other recommended guidelines, for example, breast cancer.

### Question 3: where should surveillance be offered?

It was felt by the Consensus Group that due to the expertise required in surveillance and assessment of symptoms in childhood that coordination of screening in children should managed through specialist paediatric oncology clinics.

For adults, the opinion was that coordination of screening should ideally be through clinical genetics services, provided appropriate resources where in place.

It was recognised that the ability to offer surveillance to unaffected *TP53* carriers in a non-oncology setting would be preferable but not always possible due to local arrangements.

Overall, the opinion was that a small number of national specialist clinics would be best placed to manage this work and prospectively audit the surveillance protocol. However, it was recognised that the funding of such a service may be difficult.

It was agreed that WB-MRI should only be undertaken where there is sufficient radiology expertise to report the imaging and that local clinical genetics centres should consider referral for WB-MRI to another centre if their local department were unable to work to the radiology working group standards (table 1 footnote).

## Discussion

This is the first UK clinical guideline for the surveillance and management of *TP53* carriers. The strength of the guidance is that opinion was sought prior to the meeting to identify key areas of discussion, and the Consensus Group was formed from a broad range of medical specialties and patient advocates who were given equal voice during discussion. The different perspectives and expertise of the group enriched the discussion and allowed the group to achieve a consensus view.

Our key recommendations are that a UK surveillance protocol based on international guidelines should be offered to *TP53* carriers from birth. The major limitation of the guidelines is the lack of robust evidence on which to base our discussions and recommendations. However, it was recognised that due to the rarity of LFS and *TP53* PVs, there is, and likely always will be, a limited evidence base to support screening recommendations in terms of early detection and cancer mortality. It is, therefore, recommended that discussion of the screening recommendations with patients should include a thorough discussion of the pros and cons of screening, consideration of the family history of cancer and that future screening recommendations may change as more data become available, particularly with respect to surveillance for specific variants. It is also recommended that screening data are prospectively collected and audited to inform future practice and fill gaps in our understanding.
