# Supplementary material for: UKCGG Consensus Group guidelines for the management of patients with constitutional TP53 pathogenic variants
Source: J Med Genet. 2020 Jun 22;58(2):135–9. doi: 10.1136/jmedgenet-2020-106876 (PMC7848057; doi:10.1136/jmedgenet-2020-106876)
Supplement: Supplementary data [file jmedgenet-2020-106876supp001.pdf]

Supplementary Information 1-Survey Questions

1. How many patients with a pathogenic *TP53* mutation do you have in your centre? Please indicate which centre and estimate how many children and how many adults if possible.

2. Other than breast MRI, do you currently offer any cancer surveillance to *TP53* carriers?

3. Do you agree with the International Consensus Group recommendations based on the Toronto protocol (Kratz et al 2017)

- Breast -annual MRI from 20 and consideration of risk reducing mastectomy
- Sarcoma-annual WB-MRI from birth
- ACC -3-4 monthly USS birth-18 years
- Brain-annual dedicated brain MRI from birth
- Upper GI -endoscopy 2-5yrly
- Lower GI -colonoscopy 2-5 yrly
- Skin-annual dermatology review
- General - Physical examination-3-4 monthly birth-18years and annual in adulthood

4. Does your Radiology department have the provision to offer, interpret and report Whole Body MRI?

- In adults (>18 years)
- In children (birth-18 years)

5. Do you have the provision to offer clinical review to *TP53* carriers (In children 3-4 monthly/ In adults annually)? Who would undertake clinical responsibility for this in your centre?

6. Do you currently offer predictive testing to children

7. Would cancer surveillance recommendations for children with a *TP53* mutation change your recommendations on predictive genetic testing in children?

8. In addition to *TP53* carriers, would you offer surveillance recommendations to

- Individuals meeting Classic LFS criteria?
- Individuals meeting LFL criteria? Please specify criteria?
- Individuals at 50% risk of mutation?
- Individuals with a "low penetrance mutation"?

9. If you were not able to offer local screening would you refer patients with a *TP53* mutation to a National clinic (s)?

10. Do you have any other comments or wish to raise any other questions?
